# Features of X-Band Radar Backscattering Simulation Based on the Ocean Environmental Parameters in China Offshore Seas

**DOI:** 10.3390/s18082450

**Published:** 2018-07-28

**Authors:** Tao Wu, Zhensen Wu, Jiaji Wu, Gwanggil Jeon, Liwen Ma

**Affiliations:** 1 School of Physics and Optoelectronic Engineering, Xidian University, Xi’an 710071, China; twu@stu.xidian.edu.cn; 2 School of Electronic Engineering, Xidian University, Xi’an 710071, China; wujj@mail.xidian.edu.cn (J.W.); gjeon@inu.ac.kr or ggjeon@gmail.com (G.J.); Lwma@stu.xidian.edu.cn (L.M.); 3 Department of Embedded Systems Engineering, Incheon National University, Incheon 22012, Korea

**Keywords:** China Offshore Seas, ERA-interim reanalysis data, ocean environmental parameters, finite depth sea spectrum, radar backscattering coefficient

## Abstract

The X-band marine radar has been employed as a remote sensing tool for sea state monitoring. However, there are few literatures about sea spectra considering both the wave parameters and short wind-wave spectra in China Offshore Seas, which are of theoretical and practical significance. Based on the wave parameters acquired from the European Centre for Medium-Range Weather Forecasts reanalysis data (ERA-Interim reanalysis data) during 36 months from 2015 to 2017, a finite depth sea spectrum considering both wind speeds and ocean environmental parameters is established in this study. The wave spectrum is then built into a modified two-scale model, which can be related to the ocean environmental parameters (wind speeds and wave parameters). The final results are the mean backscattering coefficients over the variety of sea states at a given wind speed. As the model predicts, the monthly maximum backscattering coefficients in different seas change slowly (within 4 dB). In addition, the differences of the backscattering coefficients in different seas are quite small during azimuthal angles of 0° to 90° and 270° to 360° with a relative error within 1.5 dB at low wind speed (5 m/s) and 2 dB at high wind speed (10 m/s). With the method in the paper, a corrected result from the experiment can be achieved based on the relative error analysis in different conditions.

## 1. Introduction

In recent years, X-band marine radar system has been employed as a remote sensing tool for sea state monitoring since it can image both the spatial and temporal variations of the sea surface with high resolutions [1,2,3]. Sea state is a qualitative and quantitative roughness of the sea surface, which can be quantified by significant wave height, wave spectrum and peak frequency [4]. The research on X-band radar backscattering coefficients based on the ocean environmental parameters is of significant importance in many fields such as ocean engineering applications and remote sensing.

Over the past decades, the offshore wave features of environmental parameters and spectra are widely studied, and one of the important issues is to predict wave parameters [5,6,7] as well as the changing climate [8], and the significant wave height can be retrieved from Doppler speed measurements through X-band marine radar [9]. Previous studies have shown that in coastal areas [6], wave system not only relates to the wind speed but also depends on the water depth, fetch, breaking waves, wave refraction and local current. In the moderated and low sea states, the wave system is often of a mixed nature, consisting of wind-sea (seas generated by the local wind) as well as swell (waves entering into the location from other areas) [10]. Empirical formulas have been developed to predict wave parameters and sea spectra in a certain area. Detail steps to predict the significant wave height in east coast of the United States were given in Andreas and Wang’s work [5]. Toba et al. [11] found that the pure wind waves follow the 3/2 exponential law as to the dominant wave period. Xu et al. [12] identified that the wind waves data achieved from a 45-year European Centre for Medium-Range Weather Forecasts reanalysis data (ERA-40) were different from Toba’s assumption, and the index of exponential law was equal to 1.17. Wang et al. developed a wind wave parameter empirical formula for coastal areas and open oceans with the basic concept of Andreas’ scheme and Toba’s exponential law, based on the observations in the northwest coast of the United States [7]. With the development of big data application, it is convenient to estimate the features in offshore sea environment according to a large number of hydrological data [13].

Currently, the real sea surface models are mainly based on the ocean wave spectra. The most commonly used wave spectrum is Pierson-Moskowitz (PM) model [14], which is valid for fully developed seas. However, the spectra are characterized with sharp peaks when the waves developed in a limited fetch, then the Joint North Sea Wave Project (JONSWAP) spectrum was proposed [15], and it has been rewritten by Goda in terms of the significant wave height and peak wave period [16], considering the influence of wave parameters. A finite depth sea spectrum based on JONSWAP spectrum was proposed by Bouws et al. [17], which has been tested with the wave data sets, but the JONSWAP spectrum does not reproduce the short waves since it is just an extended version of PM model deduced from buoy measurements, so a unified directional spectrum for long and short wind-driven waves was proposed by Elfouhaily et al. [18], which is normalized by the fundamental Cox and Munk sun glitter mean-square-slope measurements [19]. As for the China Offshore Seas, a wind wave spectrum was derived based on the existing wave energy spectra and the actual sea state by Wen et al. [20], which has been included in the Code of Hydrology for Harbor and Waterway approved by Ministry of Transport of the People’s Republic of China [21], but the spectrum was restricted to the spectral peak factor range from 1.27 to 6.77. The modified JONSWAP spectrum [16] was also adopted by the Code of Hydrology for Harbor and Waterway [21]. However, there are few literatures about sea spectra considering both the wave parameters and short wind-wave spectrum in China Offshore Seas, which are of theoretical and practical significance, and then a finite depth sea spectrum considering both wind speed and ocean wave parameters is established in this paper.

Ocean wave spectrum is important to simulate electromagnetic scattering model from sea surface. A suitable spectrum and scattering simulation model can explain some specific phenomenon in remote sensing. A unique negative upwind-crosswind asymmetry observed by L-band ocean backscatter [22] is explained by Du and Yang with a combined model [23] based on the Apel’s spectrum [24] and modified Efouhaily’s spreading function [18]. In order to study the features of sea backscattering coefficients based on the ocean environmental parameters, a suitable physical-based model is needed, and several backscattering models have been proposed, such as Wu’s improved two-scale model considering the influence of foam coverage [25], Chen and Zhang‘s semi-deterministic facet model [26] and a small-slope approximation model for Ku- and C-bands considering the breaking waves [27]. Recently Kudryavtsev et al. [28] have used the co-polarized radar geophysical model functions to retrieve short wind wave spectra.

In this paper, the features of ocean environment parameters in China Offshore Seas are analyzed based on the data acquired from ERA-Interim [29] during 36 months from 2015 to 2017, and a brief description of the wave data is presented in Section 2. Both the finite depth sea spectrum based on ocean environmental parameters and the modified two-scale model are presented in Section 3. In Section 4, the features of backscattering coefficients are investigated under monthly averaged and maximum ocean environmental parameters, wind speeds and azimuth angles. Subsequently, the conclusions are provided in Section 5.

## 2. Data Processing in China Offshore Seas

### 2.1. Brief Description of the Data and Study Area

It is well known that sea state is a qualitative and quantitative roughness of the sea surface, which can be quantified by significant wave height, wave spectrum and peak frequency [4].

**Figure 1:**
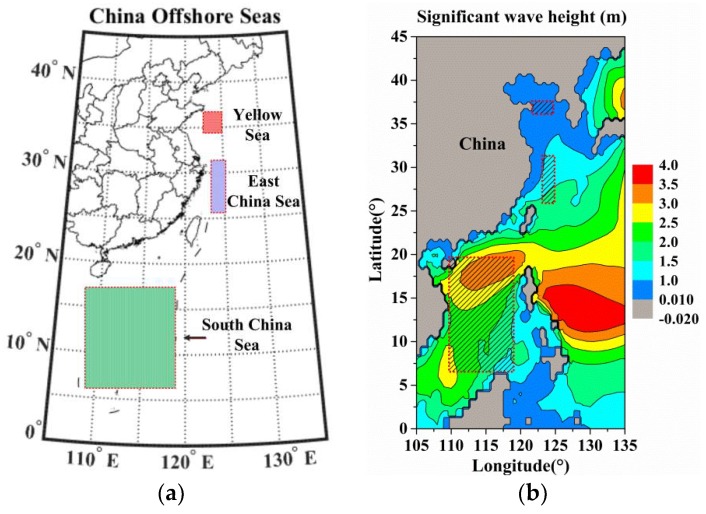
Study region of the China Offshore Seas and the sampling sea areas are marked by the dotted boxes: (**a**) The Latitude and longitude range of China Offshore Seas; (**b**) The two-dimensional (2D) color image of significant wave height data at 12:00 UTC on 1 February 2016. We can also regard the image as a 181 × 121 matrix, and the detailed data of interest are shown in Table 1.

The purpose of the present investigation is to analyze the features of wave spectra in China Offshore Seas, and to establish the relationships between wind speeds and ocean wave parameters. The offshore sea wave system is an extremely complex system influenced by geographical environment and continental shelves, the present work is based on the use of data from ERA-Interim during 36 months from 2015 to 2017. ERA-Interim is the latest global atmospheric reanalysis data produced by the European Centre for Medium-Range Weather Forecasts (ECMWF) [29]. The global spatial resolution grid is $0.25^{\circ }\times 0.25^{\circ }$ with data stored every 6h (00:00 a.m., 06:00 a.m., 12:00 a.m., and 06:00 p.m. Coordinated Universal Time (UTC)). The region of the data $\left(0\sim 45^{\circ }\;N,\;105\sim 135^{\circ }\;N\right)$ is shown in Figure 1a, and the elements of the downloaded data can be organized as a 181 × 121 matrix. For instance, the color image of significant wave height data in China Offshore Seas at 12:00 UTC on 1 February 2016 is shown in Figure 1b.

The ERA-Interim reanalysis data can provide the components of the wind vector at 10 m above the sea surface, U-component ($u_{10}$) and V-component ($v_{10}$), which can define the magnitude ($U_{10}$) and direction ($\Psi_{10}$). Equation (2) is used to achieve the wind direction ($-{180\sim 180}^{\circ }$). If the value is less than zero, just adds a constant value ($360^{\circ }$). However, the achieved wind direction is a math wind direction (md), which is measured counterclockwise from East, and the mean wave direction is a weather wind direction (wwd), which is measured clockwise from North. To convert from math wind direction to weather wind direction, Equation (3) is applied in this paper (in degrees).

The significant wave height ($H_{1/3}$), sea surface temperature (SST), mean wave period ($\bar{T}$), and mean wave direction (MWD) can also be achieved from the ERA-Interim reanalysis data:

(1) $$U_{10}=\sqrt{u_{10}^{2}+v_{10}^{2}},$$

(2) $$\Psi_{10}=\mathrm{atan}2d(v_{10},u_{10}),$$

(3) $$\mathrm{wwd}=\{\begin{array}{cc} 90^{\circ }-\mathrm{md}, & 0^{\circ }<\mathrm{md}<90^{\circ }\\ 450^{\circ }-\mathrm{md}, & 90^{\circ }<\mathrm{md}<360^{\circ } \end{array},$$

In this study, China Offshore Seas are subdivided into three sub-regions based on the latitudes (the Yellow Sea, East China Sea and South China Sea). However, the areas near the coastline are excluded because of high levels of human activities or influence of continental shelves, which can increase the uncertainties of ocean environmental data. For specific research, three sampling regions are chosen (see as the dotted boxes in Figure 1) in China Offshore Seas, the detailed information about the range of longitude and latitude grids and the corresponding elements range in the storage matrix are shown in Table 1, as well as the average depth of the study area. And there are some missing values of the wave parameter data, which have been excluded. The valid data of each single ocean environmental parameter are 22,032, 32,400, and 284,832 records for the mentioned sea areas, respectively. Then such large numbers of data are used to find the relationship between the ocean environmental parameters.

### 2.2. The Features of the Ocean Environmental Parameters in China Offshore Seas

#### 2.2.1. The Rose Map of Wind Speed and Significant Wave Height in China Offshore Seas

To obtain an understanding of the relationship between wind speeds and ocean environmental parameters, the ERA-Interim reanalysis data are first used to study the distribution of wind speeds and significant wave heights in different seas.

**Figure 2:**
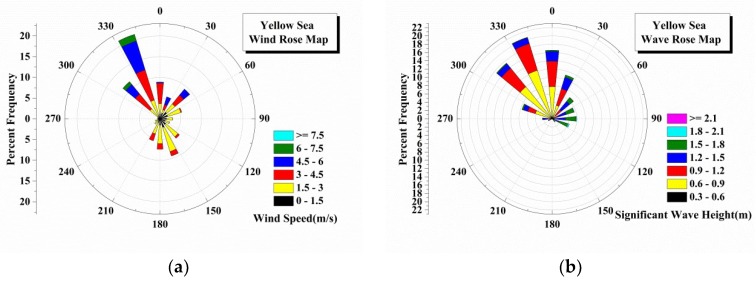

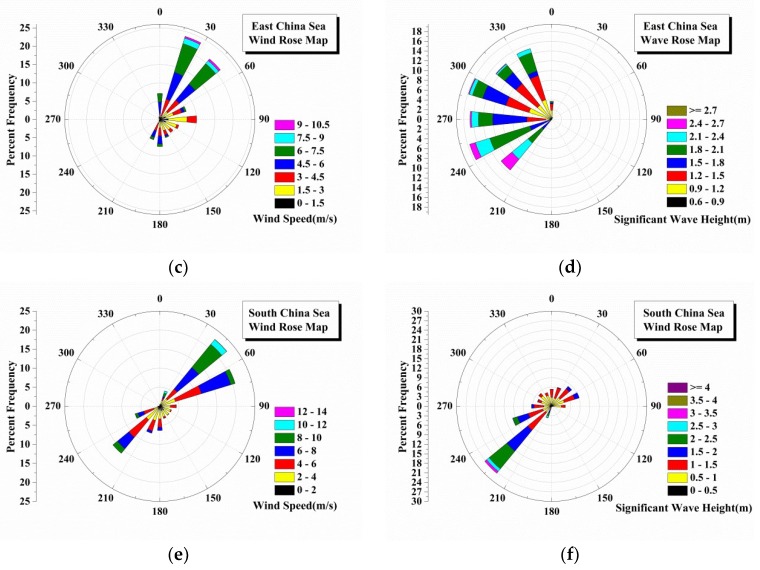
The rose map of wind speed and significant wave height in china offshore seas: Yellow Sea (**a**) Wind speed; (**b**) Significant wave height; East China Sea (**c**) Wind speed; (**d**) Significant wave height; South China Sea (**e**) Wind speed; (**f**) Significant wave height.

Figure 2 shows the polar graphic of the density of wind vector and significant wave height observed in China Offshore Seas from 2015 to 2017. From the figures, it is obviously that the mean wind directions differ from the mean wave directions except the Yellow Sea (a semi-enclosed sea), which can be due to the phenomenon of refraction [1] or the swell waves (waves entering into the location from other areas) [10]. However, most of the wave data are in the mean wave direction angle interval from 180° to 360° as show in Figure 2b,d,f, which is in contrast to the wind speed direction as shown in Figure 2a,c,e. The figures also indicate the dominant values of wind speed and wave height in different seas. The dominant wind speeds in the Yellow Sea, East China Sea and South China Sea are 3–6 m/s, 4.5–7.5 m/s and 4–10 m/s, respectively. The corresponding significant wave heights are 0.6–1.2 m, 1.2–2.4 m, and 1–2.5 m, respectively.

#### 2.2.2. Monthly Variations of the Ocean Environmental Parameters

**Figure 3:**
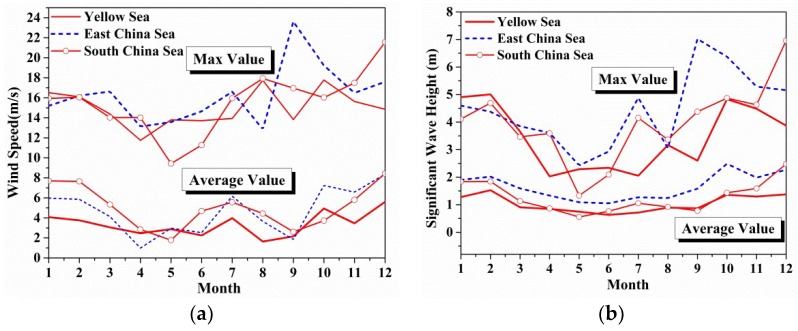

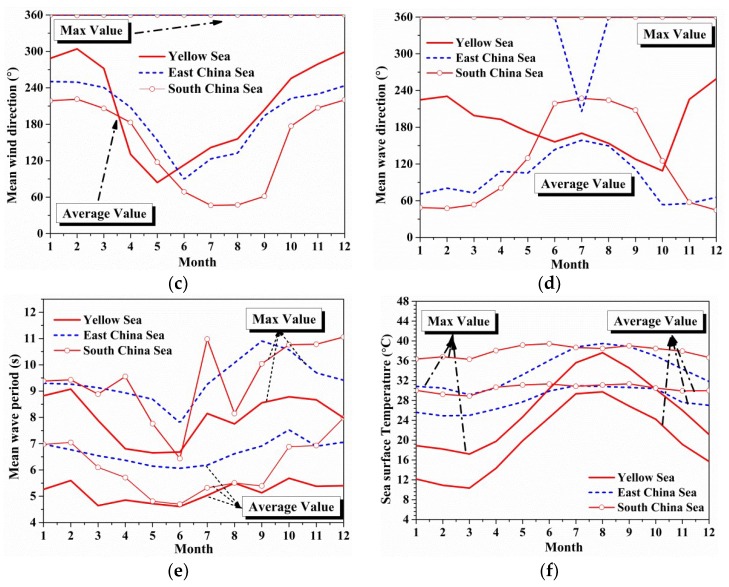
The monthly variations of averaged and maximum ocean environmental parameters in China Offshore Seas: (**a**) Wind speed; (**b**) Significant wave height; (**c**) Mean wind direction; (**d**) Mean wave direction. (**e**) Mean wave period; (**f**) Sea surface temperature.

In this section, the monthly features of ocean environmental parameters are studied averaged from 2015 to 2017, which can be considered as seasonal variation. The corresponding monthly mean backscattering coefficients can be achieved based on the following statistical data, which will be discussed in Section 4. Figure 3 gives the monthly variations of averaged and maximum ocean environmental parameters in different sea regions. From Figure 3c,d,f, seasonal characteristics can be found in mean wind direction, mean wave direction and sea surface temperature. However, the sea surface temperatures of the East China Sea and South China Sea keep relatively high all the year. From Figure 3a,b, the positive correlation between wind speed and significant wave height can be seen clearly, although the seasonal variations are small. The maximum wave direction is much lower than the others in the East China Sea in July.

#### 2.2.3. Empirical Formulas for the Relationship between the Ocean Environment Parameters

**Figure 4:**
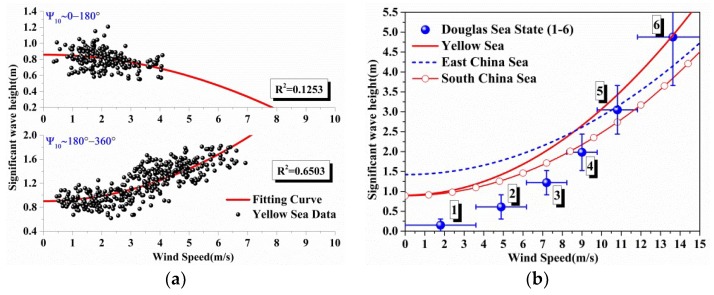
(**a**) The comparison between the scattered data (data points) based on wind direction and empirical formulas (line) versus wind speeds at above 10 m sea surface in the Yellow Sea. (**b**) Detailed comparison between empirical formulas in different seas and the Douglas Sea State, and the sea state levels from 1 to 6 are shown in the Figure 4b.

**Figure 5:**
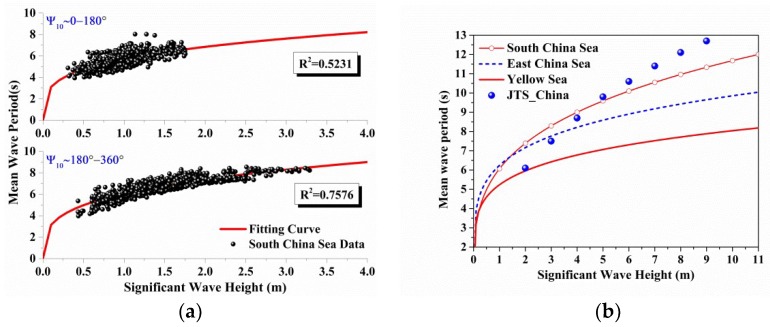
(**a**) The comparison between the scattered data (data points) based on wind direction and empirical formulas (line) versus significant wave height in the South China Sea. (**b**) Detailed comparison of the empirical formulas for China offshore seas with the approximate relation in literature [21].

Since the initial data are scattered (in Figure 4a and Figure 5a), if the mean trends of the relationship between the ocean environment parameters are required, an empirical formula is very commonly used in the ocean engineering prediction models [5,7,12].

The aim of this section is to establish the empirical formulas of the mean values for the ocean environment parameters in China Offshore Seas. Therefore, the trend of variation for the parameters in the different seas at a given wind speed can be obtained.

As shown in Section 2.2.1, most of the wave data are in a mean wave direction angle interval from 180° to 360° as show in Figure 2b,d,f, which is in contrast to the wind speed direction. The data are separated based on the wind directions since the empirical formulas are established in terms of the wind speed ($U_{10}$). It is easy to divide the wind speed data based on the sign of V component ($v_{10}$), and the corresponding wave data are used in the following statistical analysis. Although it is a simple way to separate the data, it works well as shown in Figure 4a and Figure 5a.

The empirical formula for significant wave height ($H_{1/3}$) and wind speed at 10 m above the sea surface ($U_{10}$) referred to the fitting formula reported by Wang et al. [7] can be shown as:

(4) $$H_{1/3}=a\times U_{10}^{2}+b,$$

where the coefficients of a and b are obtained by fitting to the ERA-Interim reanalysis data by means of the least-square regression.

Toba’s law [11] is applied to study the relationship between significant wave height and mean wave period, but the power index is not specified in the following empirical formula because of the existence of swell wave [12]. As the mean wave period can be achieved directly from the ERA-Interim reanalysis data, the relationship between mean wave period ($\bar{T}$) and $H_{1/3}$ can be expressed as:

(5) $$\bar{T}=a\times H_{1/3}^{b},$$

where the coefficients of a and b are obtained by fitting to the ERA-Interim reanalysis data by means of the least-square regression.

The coefficients are calculated by means of the least-square regression method with 95% confidence bounds. That is to say that the coefficients are the mean values of the 95% confidence bound values, so the empirical formulas can indicate the mean trends of the relationship between the ocean environment parameters.

To assess the performance of the empirical formulas in this study, the root mean square error (RMSE) [6] and coefficient of determination ($R^{2}$) are calculated between ERA-Interim reanalysis data and predicted values as below:

(6) $$RMSE=\sqrt{\frac{1}{n}\sum_{i=1}^{n}{\left(y_{i}-{\hat{y}}_{i}\right)}^{2}},$$

(7) $$R^{2}=\sum_{i=1}^{n}{\left({\hat{y}}_{i}-{\bar{y}}_{i}\right)}^{2}/\sum_{i=1}^{n}{\left(y_{i}-{\bar{y}}_{i}\right)}^{2},$$

where y is the observed data, $\hat{y}$ is the corresponding model predictions, $\bar{y}$ is the mean value of the observed data, and n is the total number of points. The root mean square error (RMSE) is used to measure the deviation between the observed values and model predictions. The value of $R^{2}$ varies between 0 and 1, where 1 indicates perfect agreement and 0 indicates complete disagreement.

Table 2 shows the detailed coefficients of the relationship between significant wave height and wind speed in different sea areas. It can be seen that the empirical formula for the South China Sea has the best curve fitting result ($R^{2}=0.6999$). And the proportions of the used data to total valid data (in Table 1) are both more than 50%, so the results can be considered as the trend of the relationship between significant wave height and wind speed in the different seas.

Empirical curves from least-square fits based on Equation (4) are provided for each wind speed in Figure 4a, it is obviously that the worst fitting result ($R^{2}=0.1253$) happened in the range of wind direction (0–180°), because the wave data are highly correlated to wind direction (180–360°) as shown in Figure 2a,b.

In Figure 4b, the detailed empirical formulas in different seas are compared with the Douglas Sea State model. When the sea state level is low (sea state 1 to sea state 3), the predicted values given by the proposed empirical formulas are larger than those given by Douglas Sea State model, to the author’s knowledge, it is because the component of swell waves included in the initial data, which is not related to the local wind speed but the swell wave entering into the location from other areas [10]. This figure also implies that the ocean backscattering coefficients as characterized by sea state will be different depending on whether sea state is converted to wind speed or wave height in different seas.

Table 3 shows the detailed coefficients of the relationship between significant wave height and wind speed in different sea areas. It is obviously that the empirical formula for the South China Sea has the best fitting result ($R^{2}=0.7576$) at the given wind direction (180–360°). On the contrary, the worst fitting result happens in the given wind direction (180–360°) for the East China Sea ($R^{2}=0.3607$). In Figure 2c,d (in Section 2.2.1), the initial wave data are not related to the local wind speed but the phenomenon of refraction or swell waves. So the fitting result ($R^{2}=0.6937$) at the wind direction (180–360°) will be used. The main goal in the paper is to get trend of variation for the parameters in the different seas at a given wind speed. And thus some other influence factors remain further study.

Figure 5b shows the comparison of the empirical formulas for China Offshore Seas with the approximate relation between wave height and wave period in code of hydrology for harbor and waterway [21], which can be abbreviated to JTS 145-2015. In addition, the empirical formulas are close to each other under the low wind speed (<4 m/s), and the formulas fit well to the approximate relation; while under the high wind speed (>7 m/s), the approximate relation becomes larger than the prediction values given by our empirical formulas.

## 3. Method for Electromagnetic Scattering Model

### 3.1. The Finite Depth Sea Spectrum Based on Ocean Environment Parameters

The sea state can be quantified by significant wave height, wave spectrum and peak frequency, and the purpose of this section is to establish a relationship relates ocean environment parameters to the directional wave spectrum. However, the ultimate aim is to apply this modified sea wave spectrum to the radar backscattering model in X-band which is mainly determined by the short wave roughness. A unified directional spectrum for long and short wind-driven waves proposed by Elfouhaily et al. [18] is adopted in this paper.

In order to take the water depth into consideration, a formula form based on the self-similar spectral shape (the TMA spectrum) proposed by Bouws et al. [17] is adopted, the directional spectrum in this study is expressed as the following form:

(8) $$S\left(k,\varphi \right)=\frac{1}{k}S_{E}\left(k\right)\Phi \left(k,\varphi \right)\zeta^{2}\left(kh\right),$$

where k is the wavenumber; $S_{E}\left(k\right)$ is an omnidirectional spectrum [18], Φ(k,φ) is an angular spreading function used in Elfouhaily’s work [18], $\zeta^{2}\left(kh\right)$ is a factor function considering the water depth h defined in [30].

As for the finite depth factor, a shallower coefficient introduced by McCormick [30] can be calculated according to the relative water depth ($h/L_{0}$), the function $\zeta^{2}\left(kh\right)$ is expressed as:

(9) $$\zeta^{2}\left(kh\right)=\frac{\sinh\left(2kh\right)}{\tanh\left(kh\right)\left(\sinh\left(2kh\right)+2kh\right)},$$

(10) $$kh\tanh\left(kh\right)=2\pi \cdot h/L_{0},$$

(11) $$L_{0}=\frac{g{\bar{T}}^{2}}{2\pi },$$

where the function ($\zeta^{2}\left(kh\right)$) can be calculated from the mean wave period ($\bar{T}$).

**Figure 6:**
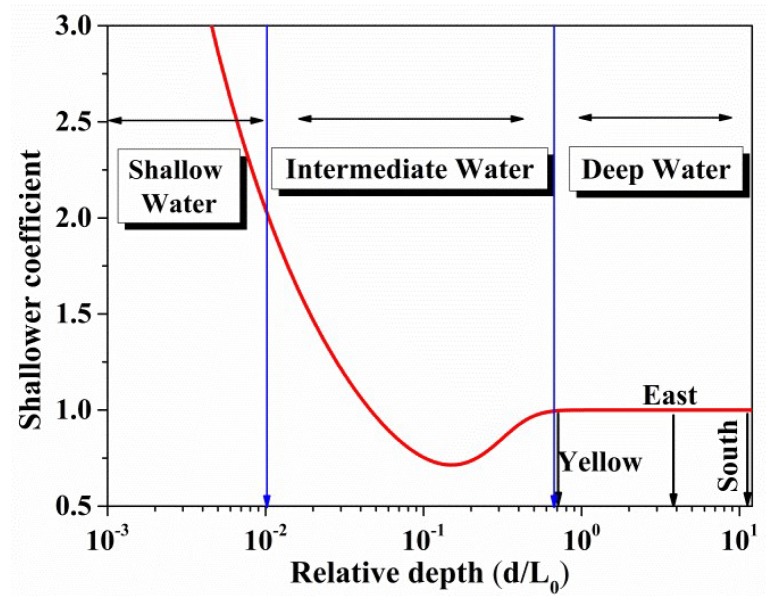
A typical example for the shallower coefficient: variation of the relative water depth for wind seed of 10 m/s. As the relative water depth increases, the shallower coefficient will be close to 1.0 for deep water.

If the wind speed ($U_{10}$) is set to 10 m/s, the mean wave period ($\bar{T}$) can be calculated by using the empirical formulas in Table 2 and Table 3. The shallower coefficient $\zeta^{2}\left(kh\right)$ as function of the relative depth is given in Figure 6. The offshore sea areas in this study can be considered as deep water. The influence of deep water on sea spectrum is mainly resulted from the wave parameters.

The original omnidirectional spectrum $S_{E}\left(k\right)$ is expressed as a sum of two spectra regimes:

(12) $$S_{E}\left(k\right)=k^{-3}\left(B_{l}+B_{s}\right),$$

(13) $$B_{l}=\frac{1}{2}\alpha_{p}\frac{c_{p}}{c}F_{p},\;B_{s}=\frac{1}{2}\alpha_{m}\frac{c_{m}}{c}F_{m},$$

(14) $$F_{p}=L_{PM}J_{p}\exp\left[-\frac{\Omega }{\sqrt{10}}\left(\sqrt{\frac{k}{k_{p}}}-1\right)\right],\;F_{m}=L_{PM}\exp\left[-\frac{1}{4}{\left(\frac{k}{k_{m}}-1\right)}^{2}\right],$$

(15) $$L_{PM}=\exp\left[-1.25{\left(k_{p}/k\right)}^{2}\right],\;J_{p}=\gamma^{\Gamma },$$

(16) $$\gamma =\{\begin{array}{cc} 1.7 & \Omega_{c}<1\\ 1.7+6\log\left(\Omega_{c}\right) & 1<\Omega_{c}<5\\ 2.7\Omega_{c}^{0.57} & \Omega_{c}>5 \end{array},$$

(17) $$\Gamma =\exp\left[\frac{{\left(\sqrt{k/k_{p}}-1\right)}^{2}}{0.08\left(1+4\Omega_{c}^{-3}\right)}\right],$$

(18) $$\alpha_{p}=0.006\Omega_{c}^{0.55},\alpha_{m}=1.4\times 10^{-2}u_{f}/c_{m},$$

(19) $$k_{p}=g\Omega_{c}^{2}/U_{10}^{2},\Omega =U_{10}/c_{p},$$

where subscripts l and s indicate long and short waves, respectively, B stands for the curvature spectrum, $c_{p}$ and $c_{m}$ are the wave phase speed. Ω and $\Omega_{c}$ are inverse wave age, and $u_{f}$ is the friction wind speed, the detail calculation process can be found in Andrea and Wang’s paper [5]. The original omnidirectional spectrum is a function of wind speed ($U_{10}$) and inverse wave age ($\Omega_{c}$). Therefore, the spectrum parameters and ocean environmental parameters should be related.

By using the ERA-40 data, Hanley and Belcher [4] have studied the global patterns of wind speed ($U_{10}$), significant wave height ($H_{1/3}$) and wave phase speed ($c_{p}$). Using the linear dispersion relation, $c_{p}$ can be calculated from the peak wave period ($T_{p}$) as:

(20) $$c_{p}=\frac{gT_{p}}{2\pi },$$

where Equation (20) is valid for deep water, $T_{p}$ can be expressed in terms of the mean wave period ($\bar{T}$) defined in Table 3 as:

(21) $$T_{p}=1.21\bar{T},$$

where Equation (21) can be found in the code of hydrology for harbor and waterway [21].

Another dimensionless inverse wave age ($\Omega_{c}$) is discussed in Elfouhaily’s work [17] for fully developed seas, and it can be combined with nondimensional fetch (X) as the following equation:

(22) $$\Omega_{c}=0.84\tanh{\left[{\left(X/X_{0}\right)}^{0.4}\right]}^{-0.75},\;X_{0}=2.2\times 10^{4},$$

where the nondimensional fetch (X) is defined as:

(23) $$X=2\times 10^{4}\times g/U_{10}^{2}.$$

As an additional check, the dimensionless significant wave height (${\tilde{H}}_{1/3}$) can be calculated as a function of dimensional fetch:

(24) $${\tilde{H}}_{s}=0.26\tanh{\left[{\left(X/X_{0}\right)}^{0.4}\right]}^{1.25},\;X_{0}=2.2\times 10^{4},$$

where ${\tilde{H}}_{s}=gH_{1/3}/U_{10}^{2}$ is referred to the model of Wilson [15]. By combining Equations (22) and (24), the dimensionless inverse wave age ($\Omega_{c}$) can be estimated by the following equation:

(25) $$\Omega_{c}=31.6938\times H_{1/3}\tanh{\left[{\left(X/X_{0}\right)}^{0.4}\right]}^{-2.0}/U_{10}^{2},X_{0}=2.2\times 10^{4}.$$

Eventually, from Equations (12)–(21) and (25), the omnidirectional spectrum ($S_{E}\left(k\right)$) can be calculated in terms of the ocean environment parameters, i.e., the wind speed ($U_{10}$), the mean wave period ($\bar{T}$) and the significant wave height ($H_{1/3}$), which can be acquired from the ERA-Interim reanalysis data.

From the above formulas, the features of sea spectrum based on ocean environment parameters are quantitatively given. The wind speed is set to 5 m/s for low sea state, and 10 m/s for high sea state. The calculated relative water depths and wave states are given in Table 4 and Table 5.

**Figure 7:**
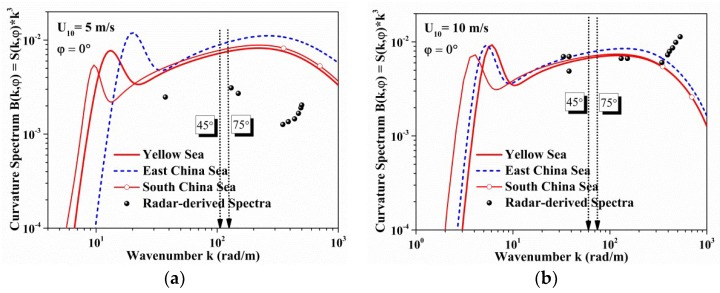
Ocean wave spectrum model considering the ocean environment parameters in different seas: (**a**) Wind speed of 5 m/s; (**b**) Wind speed of 10 m/s. (The vertical dotted lines represent the Bragg microwave wavenumber ranges, and the scattered data stand for the radar-derived spectra).

The ocean spectrum simulation based on the wavenumber ranges of Table 4 and Table 5 is shown in Figure 7, due to the rapid fall-off in omnidirectional spectrum for short waves, the features can be displayed on the curvature spectrum ($B\left(k\right)=k^{3}S\left(k\right)$). As Figure 7 shows, the spectrum energy of the Yellow Sea and South China Sea is close to each other according to the simulation based on the empirical formulas for the ocean environment parameters proposed in Table 2 and Table 3.

To verify our model, the scattered data from Kudryavtsev’s estimation [26] of short-wave spectrum from co-polarized radar backscattering cross-section are plotted in the figures. The proposed model overestimates the value in the low wind speed, and it should be infected by the components of swell waves as shown in Figure 4b in the low sea state. However, the results in high wind speed are in perfect agreement.

As we focus on the X-band marine radar, the backscattering coefficients are mainly determined by short wave roughness. The main feature of the short wave spectrum is at a Bragg microwave wavenumber ($k_{br}$), which can be calculated as:

(26) $$k_{br}=2k\sin\left(\theta \right)=\frac{4\pi \sin\left(\theta \right)}{\lambda },$$

where θ is the radar incident angle to the Bragg wave surface, λ is the wavelength, here it has the value of 0.0337 m according the X-band frequency f=8.91 GHz, the vertical lines represent the Bragg microwave wavenumbers (263.9072, and 360.5040) calculated at incident angle 45 and 75, respectively.

### 3.2. A Modified Two-Scale Model Considering the Influen of the Foam Coverage

On the real ocean surface, the Bragg short waves run along the longer surface waves which change the local incidence angles. A two scale model is usually used to simulate the sea surface scattering. This section presents a modified two-scale model (MTSM) [25] as an analytical approximate model. The backscattering coefficients calculated by MTSM model are expressed as:

(27) $$\sigma_{pp}^{0s}(\theta_{i})=Shadow(\theta_{i})\int_{-\infty }^{\infty }\;\int_{-\infty }^{\infty }{\left(\hat{p}\cdot {\hat{p}}^{\prime }\right)}^{4}c_{pp}({\theta^{\prime }}_{i},k_{i}R_{x})\cdot {\left[\sigma_{pp}^{1s}({\theta^{\prime }}_{i})+\sigma_{pp}^{2s}({\theta^{\prime }}_{i})\right]}_{R=\infty }(1+z_{x}tg\theta_{i})\cdot P(z_{x},z_{y})dz_{x}dz_{y},$$

(28) $$\sigma_{pp}^{1s}\left({\theta^{\prime }}_{i}\right)=8k_{i}^{4}\cos^{2}{\theta^{\prime }}_{i}{\left|\alpha_{pp}\right|}^{2}S(2k\sin{\theta^{\prime }}_{i},0),$$

where $\sigma_{pp}^{1s}\left({\theta^{\prime }}_{i}\right)$ is the backscattering coefficient derived from the small perturbation model, $\sigma_{pp}^{2s}\left({\theta^{\prime }}_{i}\right)$ is the additional term accounting for the surface skewness, $c_{pp}\left({\theta^{\prime }}_{i},k_{i},R_{x}\right)$ is the modified factor of curvature, $Shadow(\theta_{i})$ is the shadowing function, $P\left(Z_{x},Z_{y}\right)$ is the modified slope distribution function of the large-scale wave slopes $z_{x}$ and $z_{y}$. The symbol $\alpha_{pp}$ in Equation (27) is co-polarization coefficients, and $S\left(2k\sin{\theta^{\prime }}_{i},0\right)$ represents the modified sea spectrum in upwind defined in Section 3.1. The subscript p can be V or H which denotes the vertical polarization or the horizontal polarization.

**Figure 8:**
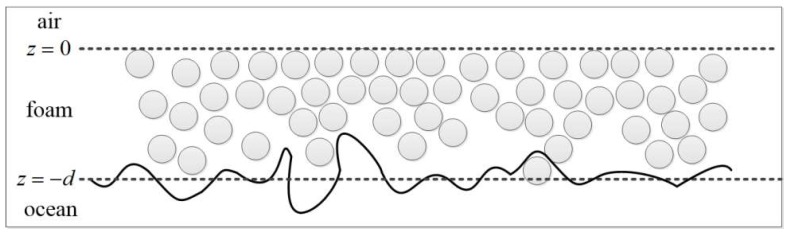
This figure shows a simple model for foam layer with foam thickness d above the rough sea surface.

The composite model is assumed to be a layer of discrete spherical foam particles above the rough sea surface according to the whitecap coverage formula (in Figure 8). And the whitecap coverage formula [31] can be express as:

(29) $$C_{w}=11.12e^{0.063U_{10}}-16.23(U_{10}\geq 7m/s).$$

From Equation (16), it is obviously that the influence of foam can be negligible in the low sea state. While the scattering in high sea state is a complicated problem, and the foam formation will contribute to microwave scattering from moderate to high wind speeds.

Reul and Chapron [32] studied the sea-foam thickness distribution, which is generated by breaking waves. They found an increase in wind speed from 7 to 20 m/s corresponding to a coverage-weighted foam-layer thickness of about 1 to 3.5 cm in unstable atmospheric conditions. The static foam-layer thickness weighted by the foam coverage and averaged over all breaking wave scales for a given wind speed can be expressed as:

(30) $$d\left(U_{10}\right)=\int \delta \cdot dF\left(U_{10},\delta \right),$$

where δ is the characteristic foam-layer thickness, $dF\left(U_{10},\delta \right)$ is the incremental static-foam fractional coverage due to the foam layers. For simplicity, the result of Equation (30) is fitting through a linear model that combines the wind speed ($U_{10}$):

(31) $$d\left(U_{10}\right)=0.000315\times U_{10}^{3.089}+0.01383\;\left(R^{2}=0.9999\right).$$

Since the coefficient of determination is very close to 1.0, Equation (31) can be considered as a good estimation formula for foam-layer thickness. Note that the wind speed is in meter per second and the foam-layer thickness is in centimeter.

As the influence of the foam layer is taken into consideration, the total backscattering coefficient is contributed by the sea surface with and without foam layer [25]:

(32) $$\sigma_{pp}(\theta_{i})=(1-C_{w})\sigma_{pp0}(\theta_{i})+C_{w}(\sigma_{pp}^{\left(0\right)}(\theta_{i})+\sigma_{pp}^{\left(1\right)}(\theta_{i})),$$

where $C_{w}$ denotes the foam coverage, $\sigma_{pp0}\left(\theta_{i}\right)$ is the backscattering coefficient of the foam-free sea surface calculated by MTSM model, $\sigma_{pp}^{\left(0\right)}\left(\theta_{i}\right)$ and $\sigma_{pp}^{\left(1\right)}\left(\theta_{i}\right)$ are the zero-order and first-order backscattering coefficients derived from the vector radiation transfer (VRT) model [31], respectively, which can be expressed as:

(33) $$\sigma_{pq}^{\left(0\right)}(\theta_{i})=4\pi \cos\theta_{i}\frac{I_{sp}^{\left(0\right)}(\theta_{i},\pi +\phi_{i})}{I_{0iq}(\pi -\theta_{i},\phi_{i})}=\sigma_{pq0}(\theta_{i})e^{-2\kappa_{e}d\sec\theta_{i}},$$

(34) $$\sigma_{hh}^{\left(1\right)}(\theta_{i})=\frac{3}{4}\cos\theta_{i}\frac{k_{s}}{k_{e}}(1-e^{-2k_{e}d\sec\theta_{i}})\cdot (1+{|R_{h0}|}^{4}\times e^{-2k_{e}d\sec\theta_{i}})+3dk_{s}{|R_{h0}|}^{2}e^{-2k_{e}d\sec\theta_{i}},$$

(35) $$\sigma_{vv}^{\left(1\right)}(\theta_{i})=\frac{3}{4}\cos\theta_{i}\frac{k_{s}}{k_{e}}(1-e^{-2k_{e}d\sec\theta_{i}})\times (1+{|R_{v0}|}^{2}e^{-2k_{e}d\sec\theta_{i}})+3dk_{s}{|R_{v0}|}^{2}e^{-2k_{e}d\sec\theta_{i}}\cos^{2}\left(2\theta_{i}\right).$$

Note that $k_{s}$ and $k_{e}$ are the scattering and extinction coefficients of foam particles; $\theta_{i}$ is the incidence angle of radar; $R_{h0}$ and $R_{v0}$ are the Fresnel reflection coefficients of flat surface for horizontal (HH) and vertical (VV) polarization, respectively.

In this paper, we mainly focus on the effects of ocean environmental parameters in sea spectrum and the corresponding sea backscattering characteristics. Note that the azimuth angle is the angle between wave direction and radar line of view.

**Figure 9:**
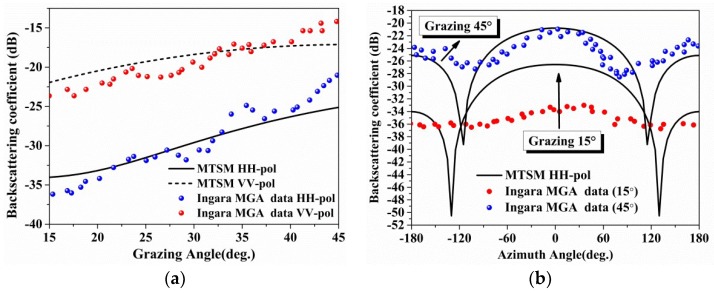
The figures show the variation of the backscattering coefficients with azimuth angle and grazing angle for MTSM Model and Ingara MGA data: (**a**) Grazing angles data and MTSM model prediction; (**b**) Azimuth angles data and MTSM model prediction.

In Figure 9, the co-polarization backscattering coefficients at X-band versus the azimuth angles and grazing angles are compared with Ingara medium grazing angles (MGA) data [33]. The measured sea state of the Ingara MGA data is reported in the literature ($U_{10}=8.5\;m/s$, $H_{1/3}=0.62\;m$ and T=3.1 s). However, the significant wave height given by the empirical formula (in Figure 4b) is much larger than what they measured. When the grazing angle locates in the region of 15–45°, as shown in Figure 9a, the simulations are very close to the data points (with a relative error less than 2 dB). However, the azimuth angles data are not in good coincidence except the results at grazing angle of 45° (only differ a lot around ±120°). The results at grazing angle of 15° differ a lot with a relative error from 2 dB to 16 dB. Guerraou and Angelliaume [33] also found a relative error up to 10 dB at grazing angle of 15° in the literature. So, the proposed scattering mode can be used in this study.

## 4. Results and Discussion

In this section, the features of X-band microwave backscattering coefficients are studied with the help of the modified two-scale model considering the influence of foam coverage. The monthly features of X-band radar backscattering coefficients based on the monthly ocean environmental parameters are calculated in Section 4.1, and then the features of backscattering coefficients under the wind speeds and azimuth angles are investigated subsequently.

### 4.1. Monthly Variation of Backscattering Coefficients Based on the Ocean Environment Parameters

The behavior of X-band backscattering coefficient is examined according to the monthly sea state parameters, i.e., the significant wave height, sea surface temperature, mean wave period, mean wave direction and wind speed.

As the monthly variations of averaged and maximum environmental parameters in different sea regions have been achieved in Section 2.2.2. The corresponding monthly X-band radar backscattering coefficients can be calculated based on the monthly ocean environment parameters in Figure 3.

**Figure 10:**
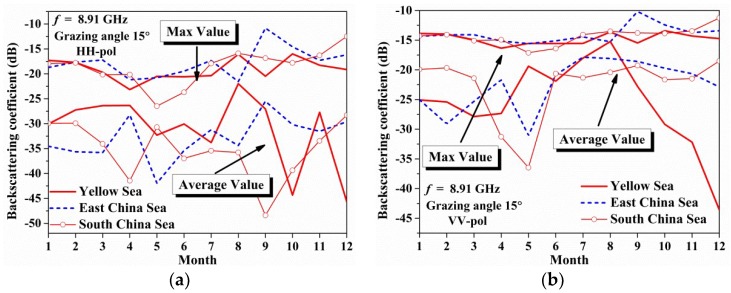
The figures show the monthly variation of the co-polarization backscattering coefficients in different seas, and both the averaged values and maximum values are plotted in the figures: (**a**) HH polarization; (**b**) VV polarization.

Figure 10 shows the monthly averaged and maximum backscattering coefficients predicted by the MTSM model in different seas. The results show that the monthly maximum coefficients in different seas change slowly (with a relative error less than 4 dB), especially in VV polarization (with a relative error less than 2 dB). But the monthly average backscattering coefficients differ a lot, and a rapid fall-off happens in the Yellow Sea from August to December in VV polarization. This is resulted from the low monthly wind speed, mean wave period and sea surface temperature.

This feature can influence the experiment measurements operated in different seas. Especially in the Yellow Sea area, a more detailed experiment should be based on the sea state parameters.

### 4.2. Mean Backscattering Coefficients Versus Wind Speed in Dfifferent Seas

In this Section, the features of mean X-band backscattering coefficients versus wind speeds in China Offshore Seas are investigated. In the calculation, sea surface temperature and salinity are set to 20 °C and 35 ‰ respectively. 

**Figure 11:**
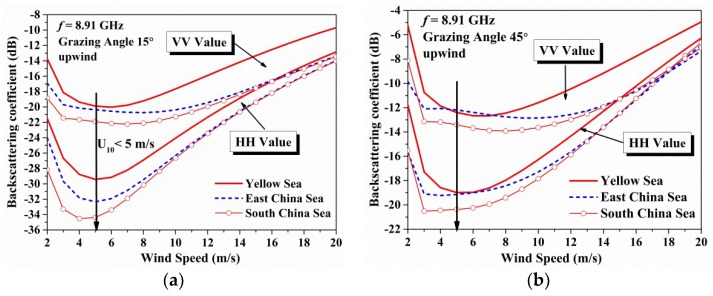
The figures show the wind speed variation of the co-polarization backscattering coefficients in different seas: (**a**) At 15° of grazing angle; (**b**) At 45° of grazing angle.

In Figure 11, the co-polarization backscattering coefficients versus the wind speeds in different seas are displayed. As the wind speed increases, the co-polarization backscattering coefficients become close to each other (within 4 dB at grazing angle of 15°, and 2 dB at grazing angle of 45°), resulting from the high significant wave heights (in Figure 4b) and increasing foam covered area based on Equation (29). When the wind speed is low (less than 5 m/s), the predicted mean backscattering coefficients are abnormally large (within 6 dB). However, this phenomenon is resulted from the influence of swell waves in the initial data in different seas. The established model is based on the ERA-interim reanalysis data, which contains the component of swell waves at low wind speed. This also indicates that the model considering both the wind speed and wave parameters work in low sea state.

### 4.3. Mean Backscattering Coefficients Versus Azimuth Angles in Different Seas

**Figure 12:**
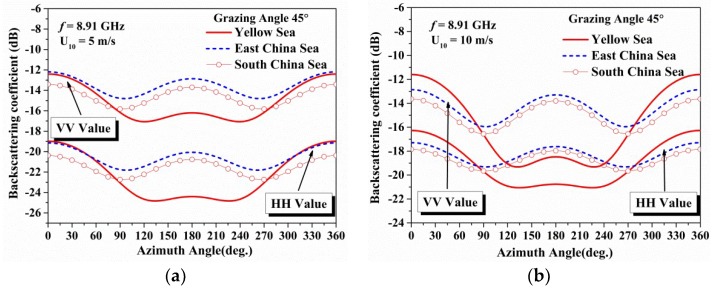
The figures show the azimuth angle variation of the co-polarization backscattering coefficients in different seas: (**a**) Wind speed of 5 m/s; (**b**) Wind speed of 10 m/s.

In this section, the features of mean X-band backscattering coefficients versus azimuth angles in China Offshore Seas are investigated. In the calculation, the grazing angle, sea surface temperature and the salinity are set to 45°, 20 °C and 35 ‰, respectively. And the remaining parameters are shown in Figure 12.

Figure 12 shows the co-polarization backscattering coefficients versus the azimuth angles in different seas. The backscattering coefficients of the East China Sea and the South China Sea are close to each other (within 1.5 dB), while the backscattering coefficients of the Yellow Sea differ from the other two seas, especially from 90° to 270°, and the coefficients are much lower than the others with a relative error up to 4 dB. In addition, the differences of the backscattering coefficients in different seas are quite small during azimuthal angles of 0° to 90° and 270° to 360° with a relative error within 1.5 dB at low wind speed (5 m/s) and 2 dB at high wind speed (10 m/s).

The model prediction can take place of experiment appropriately, which is time-consuming and expensive, if the mean trend change of backscattering coefficients is indispensable. Moreover, a modified result from the experiment can be achieved based on the error analysis in different conditions.

## 5. Conclusions

In this paper, our study relies on a single source of ERA-Interim reanalysis data in China Offshore Seas. The characteristics of ocean environment parameters in China Offshore Seas are briefly studied, using the data acquired from ERA-Interim during 36 months from 2015 to 2017. The empirical formulas for the relationships of significant wave height, wind speed and mean wave period are also given. The ocean environmental parameters are put into a finite depth sea spectrum and sea backscattering simulation model, so as to investigate the backscattering coefficients features of China Offshore Seas in different sea conditions. Hence, the final results are the mean backscattering coefficients over the variety of sea states at a given wind speed.

The values of sea spectra in different seas become close in high wind speeds as the model predicted in this paper. The results show that the monthly maximum coefficients in different seas change slowly (within 4 dB), especially in VV polarization. As for the low sea state, an abnormally large value compared with the high sea state is found since the component of swell waves at low wind speed is considered. In addition, the differences of the backscattering coefficients in different seas are quite small during azimuthal angles of 0° to 90° and 270° to 360° with a relative error within 1.5 dB at low wind speed (5 m/s) and 2 dB at high wind speed (10 m/s). Since the relative error in different seas is analyzed, the model prediction can take place of experiment appropriately, which is time-consuming and expensive. Moreover, a correction result after the experiment can be achieved based on the error analysis in different sea condition.

The wind speed is a leading factor in ocean environment, and it also plays an important role in the backscattering coefficients model. However, in the moderated and low sea states, the sea wave system is often of a mixed nature, consisting of wind-sea as well as swell. So it is appropriate to establish a two-peak spectrum, but there are few two-peak spectra considering the short wave spectra, which remain to be solved in the future.

## Figures and Tables

**Table 1:** The range of longitude and latitude grids of study areas and the corresponding data ranges.

Sea Areas	Average Depth (m)	Latitude Range	Longitude Range	Matrix Row	Matrix Column	Valid Data
Yellow Sea	44	34–36	121–125	37–45	65–81	22,032
East China Sea	370	24–30	123–125	61–85	73–81	32,400
South China Sea	1212	6–18	109–119	109–157	17–57	284,832

**Table 2:** The coefficient and performance of empirical formulas in the different seas for Equation (4).

Sea Areas	Empirical Formula				Wind Direction (Degree)	Used Data (Proportion)
	*a*	*b*	*RMSE*	*R*^2^		
Yellow Sea	0.02163	0.9023	0.1698	0.6503	180~360	14,259 (64.72%)
East China Sea	0.0147	1.421	0.2175	0.6273	180~360	21,482 (66.30%)
South China Sea	0.01585	0.8895	0.3113	0.6999	180~360	147,571 (51.81%)

**Table 3:** The coefficient and performance of empirical formulas in the different seas for Equation (5).

Sea Areas	Wind Direction $(0\sim 180^{\circ })$				Wind Direction $(180\sim 360^{\circ })$			
	*a*	*b*	*RMSE*	*R*^2^	*a*	*b*	*RMSE*	*R*^2^
Yellow Sea	5.823	0.4848	0.4429	0.4217	5.233	0.1866	0.2909	0.4178
East China Sea	5.883	0.4273	0.4174	0.6937	6.242	0.1983	0.3689	0.3607
South China Sea	5.704	0.2631	0.4707	0.5231	6.065	0.2846	0.4152	0.7576

**Table 4:** The relative water depth and wave parameters for wind speed of 5 m/s.

Sea Areas	Depth (m)	*H*_1/3_ (m)	*T_p_* (s)	Relative Water Depth (*h*/*L*_0_)	Inverse Wave Age
Yellow Sea	44	1.4431	6.7804	0.8974	0.4723
East China Sea	370	1.7885	8.4757	4.8298	0.3778
South China Sea	1212	1.2858	7.8828	18.2903	0.4063

**Table 5:** The relative water depth and wave parameters for wind speed of 10 m/s.

Sea Areas	Depth (m)	*H*_1/3_ (m)	*T_p_* (s)	Relative Water Depth (*h*/*L*_0_)	Inverse Wave Age
Yellow Sea	44	3.0653	7.8039	0.6775	0.8207
East China Sea	370	2.8910	9.3225	3.9922	0.6870
South China Sea	1212	2.4745	9.4973	12.6002	0.6743
